# Hepatic iron overload is associated with hepatocyte apoptosis during *Clonorchis sinensis* infection

**DOI:** 10.1186/s12879-017-2630-3

**Published:** 2017-08-01

**Authors:** Su Han, Qiaoran Tang, Rui Chen, Yihong Li, Jing Shu, Xiaoli Zhang

**Affiliations:** 1 Department of Parasitology, Harbin Medical University, Harbin, 150081 China; 2 Department of Orthopaedic, The fourth Affiliated Hospital of Harbin Medical University, Harbin, China

**Keywords:** *Clonorchis sinensis*, Iron, Apoptosis, Infection, Patients

## Abstract

**Background:**

Hepatic iron overload has been implicated in many liver diseases; however, whether it is involved in clonorchiasis remains unknown. The purpose of this study is to investigate whether *Clonorchis sinensis* (*C. sinensis*) infection causes hepatic iron overload, analyze the relationship between the iron overload and associated cell apoptosis, so as to determine the role of excess iron plays in *C. sinensis*-induced liver injury.

**Methods:**

The Perls’ Prussian staining and atomic absorption spectrometry methods were used to investigate the iron overload in hepatic sections of wistar rats and patients infected with *C. sinensis*. The hepatic apoptosis was detected by transferase uridyl nick end labeling (TUNEL) methods. Spearman analysis was used for determining the correlation of the histological hepatic iron index and the apoptotic index.

**Results:**

Blue iron particles were deposited mainly in the hepatocytes, Kupffer cells and endothelial cells, around the liver portal and central vein area of both patients and rats. The total iron score was found to be higher in the infected groups than the respective control from 8 weeks. The hepatic iron concentration was also significantly higher in treatment groups than in control rats from 8 weeks. The hepatocyte apoptosis was found to be significantly higher in the portal area of the liver tissue and around the central vein. However, spearman’s rank correlation coefficient revealed that there was a mildly negative correlation between the iron index and hepatocyte apoptosis.

**Conclusions:**

This present study confirmed that hepatic iron overload was found during *C. sinensis* infection. This suggests that iron overload may be associated with hepatocyte apoptosis and involved in liver injury during *C. sinensis* infection. Further studies are needed to investigate the molecular mechanism involved here.

## Background

Clonorchiasis, caused by *Clonorchis sinensis* (*C. sinensis*), is an important food-borne parasitic disease. It is estimated that 35 million people are infected worldwide, including approximately 15 million people in China [1]. Humans become infected by ingesting freshwater fish containing *C. sinensis* metacercariae. Clonorchiasis remains a significant public health problem in China. Heavy or long-term chronic infections provoke epithelial hyperplasia, periductal fibrosis, cholelithiases, cholecystitis, hepatic fibrosis, and even cholangiocarcinoma [2]. In addition, *C. sinensis* has been classified by the International Agency for Research on Cancer (IARC) as a group 1 biocarcinogen in humans [3]. The main mechanism for hepatic and biliary damage is that the flukes migrate through the biliary system and they secrete or excrete metabolic products, which result in immunopathogenesis and simultaneous liver injury [4, 5]. However, the mechanism underlying hepatic and biliary damages in hosts has yet to be fully elucidated.

Iron is a vital requirement for normal cellular physiology. Liver is a major regulator of body iron metabolism. Iron stored within ferritin is thought to be bioavailable. Hepcidin, a liver-produced peptide, regulates body iron homeostasis, by controlling cellular efflux of iron from enterocytes, hepatocytes, and macrophages. Disturbances of iron homeostasis commonly is manifest in inflammatory and infectious diseases [6]. Iron overload leads to the generation of reactive oxygen species (ROS) and lipid peroxidation by the Fenton reaction, which causes oxidative damage to DNA and abnormal protein expression, and subsequently induces damage to mitochondria and lysosomes [7]. Iron overload could induce the apoptosis or necrosis of hepatocytes via oxidative stress [8]. It is also possible that iron overload could promote the production of collagen by activated hepatic stellate cells and participate in the development of liver fibrosis [9]. Therefore, iron overload is an initiator of hepatocyte apoptosis or liver injury. Iron overload associated with liver diseases has received increasing levels of attention. In addition, abnormal iron metabolism has been reported in some parasitic diseases, such as schistosomiasis and toxoplasmosis [10]. However, iron metabolism in *C. sinensis*-infected liver tissue has not been reported until now.

In this study, we detected iron deposition and hepatocyte apoptosis in the liver of patients and rats infected with *C. sinensis.* The aim was to determine whether hepatic iron overload is involved in clonorchiasis, and to analyze the relationship between the iron overload and associated cell apoptosis, so as to determine the role of excess iron in *C. sinensis*-induced liver injury.

## Methods

### Preparation of *C.sinensis* metacercariae

*C.sinensis* metacercariae (MC) were isolated from the second intermediate host, *Pseudorasbora parva* captured in the Songhuajiang River, where is an endemic area of *C.sinensis* infection in China. *C. sinensis* MC were found with similar morphology from different trematode, such as *Opisthorchis spp*., and some groups of minute intestinal flukes (Heterophyidae, including *Metagonimus yokogawai, M. takahashii, Stellantchasmus falcatus, Centrocestus formosanus, Haplorchis pumilio, H. taichui, and H. yokogawai*) [11, 12]. Except from *C. sinensis*, there is no evidence that above fish-borne trematode infections with similar morphology are endemic in Heilongjiang Province [13–15]. Thus, there is no difficulty in differential diagnosis between *C.sinensis* and other trematode with similar morphology.

Metacercariae were collected as described previously [16]. Briefly, the fishes were digested with zcid pepsin solution (0.2% HCL, 0.6% pepsin, PH 2.0) and isolated under stereomicroscope. *C.sinensis* MC were kept in 0.1 M phosphate-buffered salina (PBS, PH 7.4) at 4 °C until using for amino model. In addition, we made a subsequent study lab-tested (PCR identification) and confirmed the species as *C.sinensis* (the results were shown in another unpublished paper).

### Experimental animals and design

Wistar rats at 5–6 weeks of age were purchased from the animal center of Harbin Medical University. All the rats were randomly divided into 2 groups, infection group and control group. Each group consisted of 16 rats and was kept in the clean animal cages until killed. In the infection group, the rats were infected 100 *C.sinensis* MC, and killed by ether anesthesia at 4, 8, 12, and 16 weeks post infection. Control group rats were mock-infected with saline. Parasitic infection was successfully identified by detecting the *C.sinensis* eggs in the fecal samples of rats. At different time points after parasite infection, livers were extirpated after rats killed by ether anesthesia. The liver tissues were fixed in 4% formalin for 24 h prior to embedding in paraffin wax and sectioning.

### Collection of the patients with clonorchiasis

Autopsy specimens of four patients who died from other diseases infected with *C. sinensis,* and four normal human liver specimens from traffic accident as controls were retrieved from the Department of Forensic Medicine, Harbin Medical University between 2000 and 2007. In addition, these four patients with *C. sinensis* infection did not have other liver diseases. All subjects were enrolled with written informed consent and Ethics Committees of Harbin Medical University approval. Autopsy tissue samples, demographic, clinical, and laboratory data were collected and coded to protect participants’ confidentiality in accordance with approved protocols at Harbin Medical University.

### TUNEL assay

To detect apoptosis in human and rat liver tissues, an Apoptosis Detection Kit (Roche, Germany) was used to carry out TUNEL method on 4-μm-thick paraffin sections according to the manufacturer’s instructions, the sections were visualized with 3, 3-diaminobenzidine tetrahydrochloride and counterstained with Gill’s hematoxylin. Positive cells and total hepatocytes were counted from 20 randomly selected high power fields (×400) from each section under light microscopy and the positive rates of TUNEL were calculated.

### Histological staining of iron

Hepatic iron accumulation was performed using the Perl’s Prussian blue staining method [17]. Briefly, liver sections were deparaffinized and rehydrated by the standard procedure. Slides were incubated in Perls’ Staining Solution (comprising equal parts of potassium ferrocyanide and HCL) for 20 min. Then, the sections were washed with Milli-Q water and stained with nuclear fast Red for 5–10 min, dehydrated, cleared in xylem and mounted using a standard procedure. Deposits of iron were stained as blue by Perl’s Prussian blue staining, while cytoplasm and cellular nucleus were stained as pink and red, respectively.

### Histological iron index

The degree of hepatic iron accumulation was assessed by histological hepatic iron index (HHII), according to both their amount and their cellular and lobular location in Rappaport’s acinus described by Deugnier et al. [18]. Histological iron index was grading by three different iron scores: sinusoidal iron score (SIS;0–12), portal iron score (PIS;0–12), hepatocytic iron score (HIS;0–36). The sum of these scores was defined as the total iron score (TIS; range, 0–60).

### Determination of hepatic tissue iron concentration

Hepatic iron concentrations (HIC) were measured by atomic absorption spectrometry as described previously [19], and expressed as micrograms non-heme iron per gram of wet tissue weight. Briefly, about 0.5 g of liver samples were wet digested by 15 ml configured mixed acid (HClO_4_: HNO_3_ = 1:4), cold digestion overnight, and heating by electric hot plate. After digestion, samples were resuspended in 0.1 mol/L of nitric acid to a final volume of 10 ml. The concentration of iron in the samples was analyzed for non-heme iron by flame atomic absorption spectrophotometer (DongXi Electronic, Beijing, China).

### Statistical analysis

Dates were presented as mean ± SE according to variables distribution. The relationship between the iron deposition, and apoptosis was analyzed using a 2-sided Spearman test. The difference between the non-parametric independent variables was tested according to Mann-Whitney U-tests. SPSS version 13.0 was performed for all statistical analysis. *P* < 0.05 was considered as significant.

## Results

### Histological evaluation of iron deposits in the livers of patients infected with *C. sinensis*

**Fig. 1:**
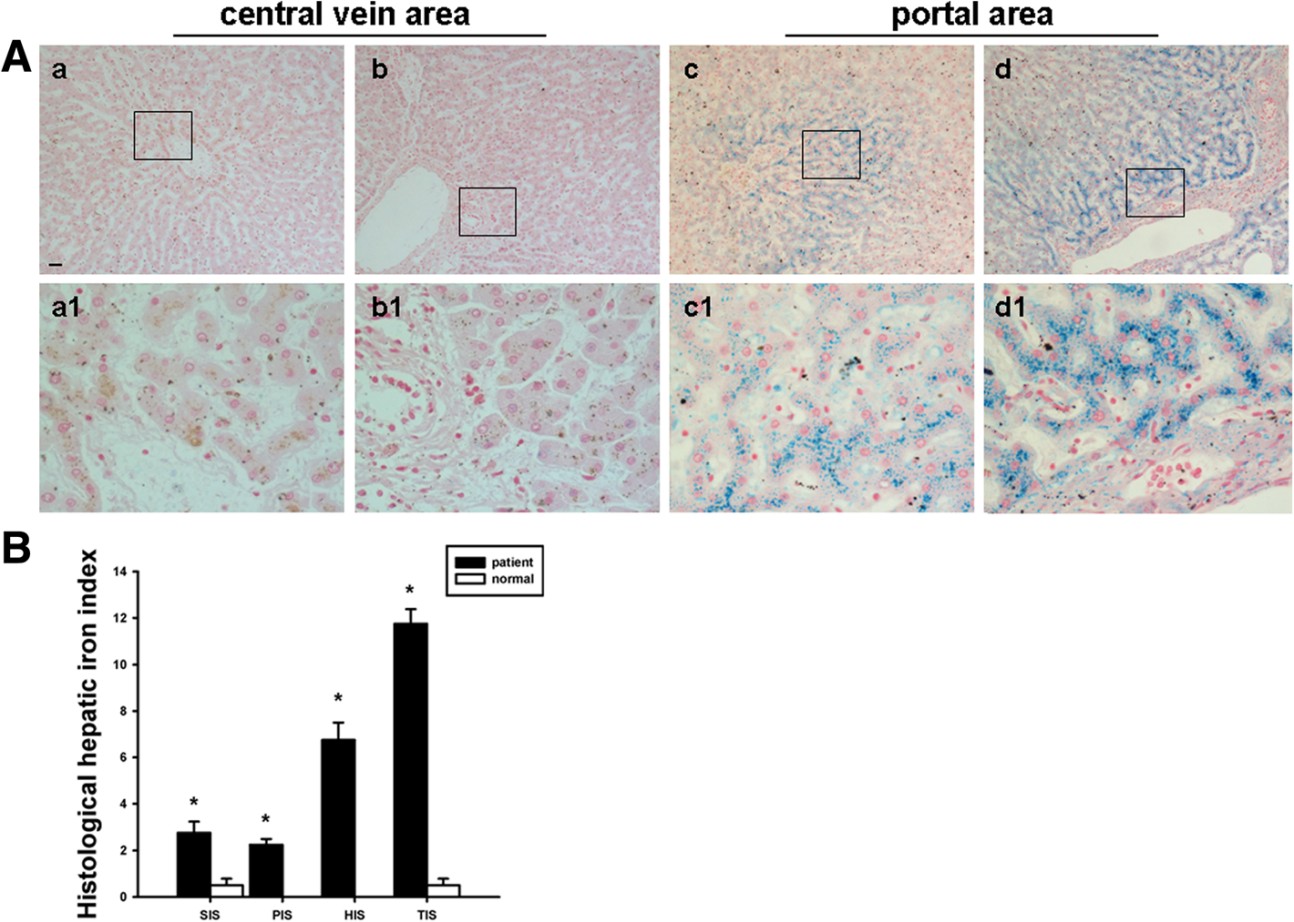
Assessment of hepatic iron deposition in patients with *Clonorchis sinensis* infection. **A** Iron deposits were found in liver tissue from patients (*c-d*) infected with *C. sinensis,* whereas none were recorded in normal controls (*a-b*). Original magnification; ×100; high power fields: ×400; Scale bar: 50 μm. **B** Total iron score (TIS) was significantly higher in patients infected with *C. sinensis* than in normal control, using Histological iron index. SIS, sinusoidal iron score; PIS, portal iron score; HIS, hepatocytic iron score. * *p* < 0.05

Intracellular iron deposits were detected by Prussian blue staining. Deposits were found in liver tissue from patients infected with *C. sinensis,* whereas none were recorded in normal controls (Fig. 1A). However, the deposits of iron were not homogeneous. In Rappaport’s acinus, iron deposits were seen as large or small granules, and sometimes as a confluent mass of granules. Iron deposits were found distributed in hepatocytes, Kupffer cells and endothelial cells, and in a decreasing gradient from the periportal area to the perivenous area. Hepatocytes with iron depositions were found to be severely damaged.

Iron deposits were assessed according to both the quantity and cellular and lobular location in Rappaport’s acinus. It revealed that the TIS was significantly higher in patients infected with *C. sinensis* than in normal control (*p* < 0.05) (Fig. 1B).

### Histological evaluation of iron deposits in the livers of rats infected with *C. sinensis*

Intracellular iron deposits were observed to varying degrees in the livers of *C. sinensis*-infected rats, whereas results were consistently negative in control rats. Large free iron deposits, visible as dark, deep-blue inclusions, were observed in Rappaport’s acinus. The quantity of iron deposits was significantly lower in rats than in patients, and the staining of iron granules was also weaker in rats.

**Fig. 2:**
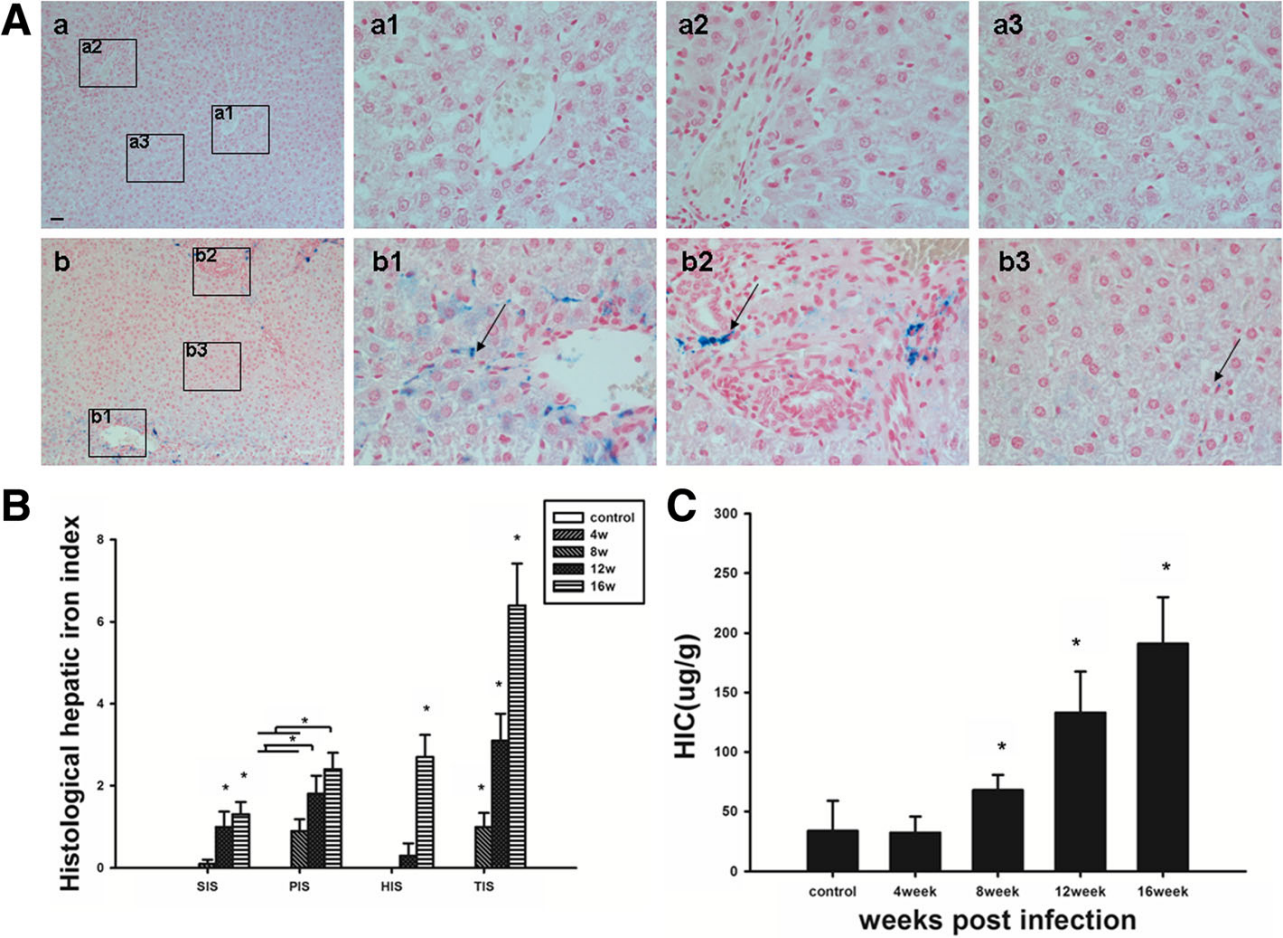
Assessment of hepatic iron deposition in rats with *Clonorchis sinensis* infection. **A** Histological staining of hepatic iron deposition of control (a × 100), (a1-a3 × 400), and infected rats for 16 weeks (b × 100), (b1-b3 × 400). Arrows showed iron. Scale bar: 50 μm. **B** Total iron score (TIS) of infected groups were significantly higher than those of control groups, using Perl’s Prussian blue staining, at 8, 12 and 16 weeks. *p < 0.05 **C**. Hepatic iron concentration (HIC) was significantly higher at 8, 12 and 16 weeks after *C. sinensis* infection compared to the control groups. *p < 0.05

Iron deposits were found within Kupffer and endothelial cells, and to a lesser extent, within portal macrophages. Iron deposits were predominantly in the portal and central vein areas (Fig. 2A).

### Assessment of iron deposition in the liver of rats

Further histological evaluation revealed that iron deposits were apparent from 8 weeks after infection in rat liver tissue. TIS of infected group were significantly higher than those of control groups, at 8, 12 and 16 weeks (*p* < 0.05) (Fig. 2B).

Atomic absorption was used to quantify the level of accumulated iron in liver tissue. This revealed that the HIC was significantly higher at 8, 12 and 16 weeks after *C. sinensis* infection compared to the control groups (*p* < 0.05) (Fig. 2C).

### Apoptosis in patients and rats after *C. sinensis* infection

**Fig. 3:**
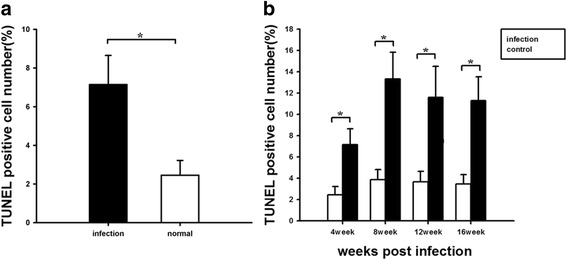
Evaluation of apoptosis of liver with *Clonorchis sinensis* infection. The apoptosis of liver in patients (**a**) and rats (**b**) with *Clonorchis sinensis* infection. * *p* < 0.05

We found that the cell apoptotic index was significantly higher in patients with *C. sinensis* infection than in the control groups (Fig. 3a). Furthermore, we determined that the hepatocyte apoptotic index of rats with *C. sinensis* infection increased from 4 weeks post-infection, and reached a peak at 8 weeks (Fig. 3b).

### The correlation between iron deposition and the apoptotic index

**Fig. 4:**
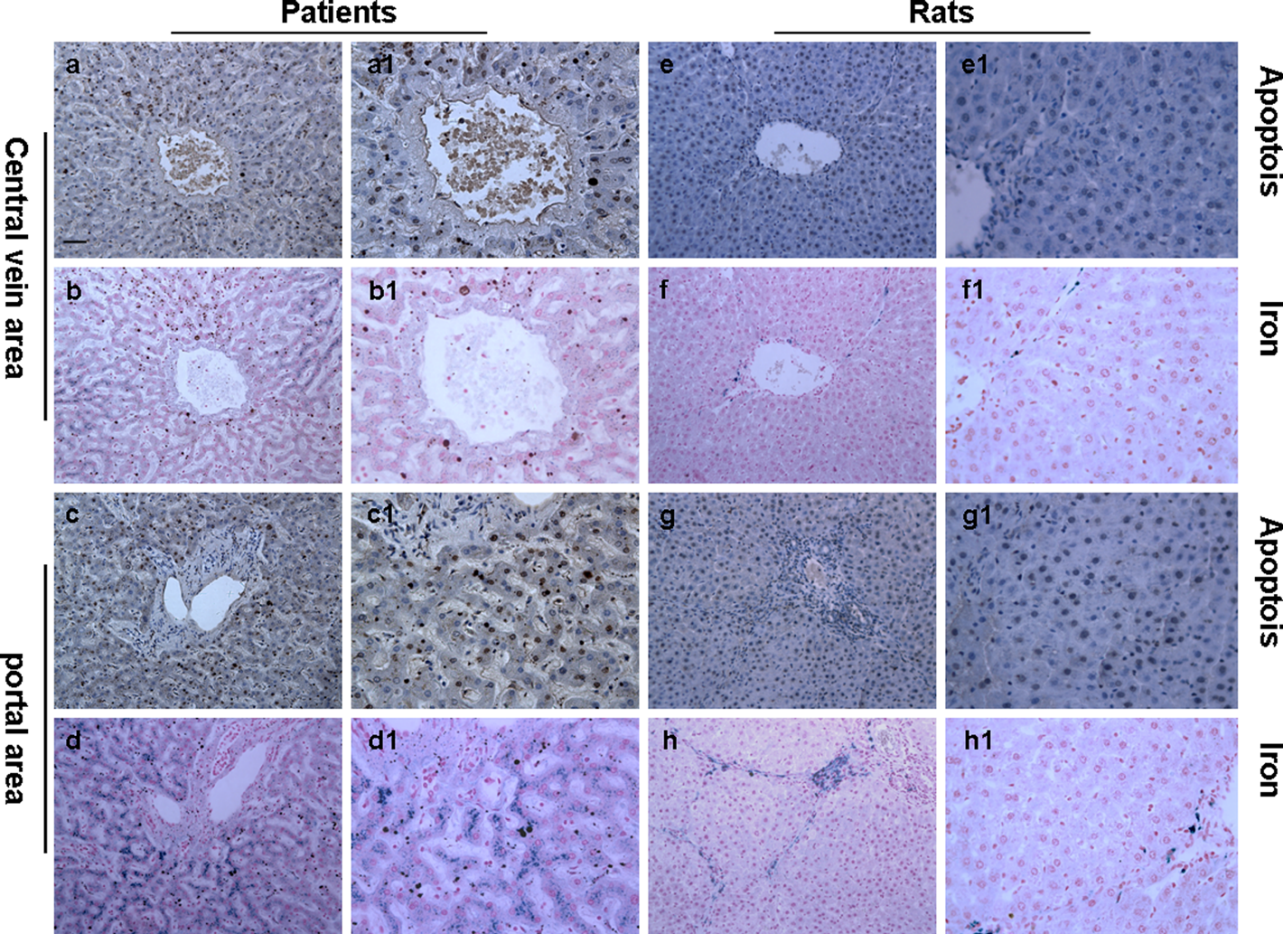
Assessment of the correlation between hepatic iron deposition and the apoptosis. Hepatic iron deposition and the apoptosis were mainly distributed around the central vein and portal areas in the liver. Original magnification; ×200; high power fields: ×400; Scale bar: 50 μm

We found that the hepatocytes staining positively with the TUNEL assay were mainly distributed around the central vein and portal area in the liver. Similarly, iron particle deposition within hepatocytes, Kupffer cells and epithelial cells was found to be distributed around the central vein and portal areas (Fig. 4). Analysis using Spearman’s rank correlation coefficient revealed that there was a mildly negative correlation between the TIS and the apoptotic index in rats infected with *C. sinensis* (*r* = −0.112, *P* > 0.05).

## Discussion

Studying iron metabolism has provided information regarding the pathogenic mechanisms of various diseases. In chronic virus hepatitis, drug-induced liver injury and alcoholic liver disease, disturbed iron homeostasis and body iron overload are common [20, 21]. Hepatic iron deposition can be detected using Prussian blue staining and atomic absorption spectrometry methods. Prussian blue staining is a highly effective imaging technique and enables the distribution of iron in different cells to be determined, while the highly sensitive method of atomic absorption spectrometry detects low levels of iron deposition in the liver [22]. In our study, hepatic iron deposition was detected by combining the two methods. Our results showed that iron deposition was found in the liver with *C. sinensis* infection.

The iron deposition was found predominantly in periportal hepatocytes (zone 1) and perivenous hepatocytes (zone 3), while the lowest levels of deposition were found in the zone 2 area. This distribution pattern was consistent with that seen in chronic viral hepatitis and alcoholic liver disease [23], and may be relate to the functional status of the cells or to the differential expression of iron transporters that may facilitate periportal hepatocyte iron loading. Furthermore, based on their localization within differing zones of the liver acinus, the hepatocytes, Kupffer cells, and sinusoidal endothelial cells have been reported to demonstrate different metabolic capabilities. Iron is absorbed from the intestine and delivered to the liver via the portal vein, suggesting that hepatocytes in close proximity to the portal vein (periportal hepatocytes) would be loaded preferentially [7].

With respect to the mechanism of iron overload, genetic mutations may be involved, for example in *HFE* and *TRF2* genes, which are associated with iron-overload diseases [7]. However, it has also been suggested that factors such as alcoholism and hepatitis C virus play a significant role. It is possible that such factors could result in a deficiency in hepcidin induction, resulting in iron being absorbed by intestinal cells. On the other hand, iron accumulation in Kupffer cells has been attributed to the phagocytosis of necrotic/apoptotic hepatocytes, which absorbed ferritin and hemosiderin from damaged hepatocytes [7, 24]. In summary, we speculated that liver iron overload in *C. sinensis* infection may be associated with abnormal iron metabolism, thus increasing the amount of iron absorbed, and with elevated levels of iron being released from damaged hepatocytes or through the phagocytosis of damaged, iron-loaded hepatocytes by Kupffer cells.

In our previous study, we had confirmed that *C. sinensis* infection could induce hepatocyte apoptosis [16]. It has been demonstrated previously that the Fas/FasL system, via caspase-3 activation, plays a key role in *C. sinensis*-infected hepatocyte apoptosis [16]. However, the systems involved in apoptosis of cells within the parasite-infected liver are complex, and additional investigation will be needed to elucidate the precise pathways. In this study, we analyzed the relationship between the apoptosis of hepatocytes and iron overload. Iron and apoptosis were also around the central vein and portal areas in the liver. It suggested that the apoptosis of hepatocytes might be induced directly by unknown factors released by *C. sinensis*, such as excretory-secretory antigen produced by the liver fluke, or indirectly through the inflammatory cells that contribute to liver tissue damage.

This study showed that the distribution of iron was consistent with the location of hepatocyte apoptosis following *C. sinensis* infection. It has been shown that iron overload resulted in the production of ROS and lipid peroxidation, which was thought to contribute to hepatocyte necrosis and apoptosis [7]. Iron is required for the initial steps of Fas-mediated apoptosis and caspase activation, which aggravated tissue injury by increasing apoptosis [25, 26]. The elevated iron activated caspase3 via enhanced oxidative stress contributes to hepatic apoptosis and the development of liver injury [27]. Therefore, it is suspected that iron overload promotes the formation of ROS and lipid peroxidation, which subsequently acting as markers of apoptosis or participated in caspase-3-mediated hepatocyte apoptosis in *C. sinensis* infection. Furthermore, hepatic iron deposition might increase Kupffer cell iron levels, affect the host’s immune surveillance, immune regulation, inflammatory response and cytokine activity, and thus participate in the pathogenic response to *C. sinensis* infection. In other words, there may be a correlation between the level of iron deposition (TIS) and the apoptotic index. However, we found that there was a mildly negative correlation between the iron index and hepatic apoptosis. Because we deleted the samples (TIS = 0) and just analysis 24 rats with *C. sinensis* infection, it was possible that the mildly negative correlation may be related with the smaller numbers of samples. Thus, in the future study, we should enlarge the number of samples, in order to analysis the statistically correlation.

## Conclusions

Our results demonstrate that iron overload in the liver is associated with *C. sinensis* infection. The iron overload is associated with hepatocyte apoptosis and involved in liver injury during *C. sinensis* infection, which plays a significant role in clonorchiasis. However, there are some issues need to be explored. Why is the iron content high in *C. sinensis* infection? What is the molecular mechanism involved here? Is it related to disruption of iron regulation or some other mechanism? These concepts are needed to investigate in the further study.

## Abbreviations

*C. sinensis*: *Clonorchis sinensis*
ESPs: Excretory-secretory products
HIC: Hepatic iron concentration
HIS: Hepatocytic iron score
PIS: Portal iron score
ROS: Reactive oxygen species
SIS: Sinusoidal iron score
TIS: Total iron score
TUNEL: Transferase uridyl nick end labeling
